# Days’ Supply of Initial Opioid Analgesic Prescriptions and Additional Fills for Acute Pain Conditions Treated in the Primary Care Setting — United States, 2014

**DOI:** 10.15585/mmwr.mm6806a3

**Published:** 2019-02-15

**Authors:** Mallika L. Mundkur, Jessica M. Franklin, Younathan Abdia, Krista F. Huybrechts, Elisabetta Patorno, Joshua J. Gagne, Tamra E. Meyer, Judy Staffa, Brian T. Bateman

**Affiliations:** ^1^Office of Surveillance and Epidemiology, Center for Drug Evaluation and Research, Food and Drug Administration, Silver Spring, Maryland; ^2^Division of Pharmacoepidemiology and Pharmacoeconomics, Department of Medicine, Brigham and Women’s Hospital and Harvard Medical School, Boston, Massachusetts.

During 2017, opioids were associated with 47,600 deaths in the United States, approximately one third of which involved a prescription opioid (*1*). Amid concerns that overprescribing to patients with acute pain remains an essential factor underlying misuse, abuse, diversion, and unintentional overdose, several states have restricted opioid analgesic prescribing (*2*,*3*). To characterize patterns of opioid analgesic use for acute pain in primary care settings before the widespread implementation of limits on opioid prescribing (*2*,*3*), patients filling an opioid analgesic prescription for acute pain were identified from a 2014 database of commercial claims. Using a logistic generalized additive model, the probability of obtaining a refill was estimated as a function of the initial number of days supplied. Among 176,607 patients with a primary care visit associated with an acute pain complaint, 7.6% filled an opioid analgesic prescription. Among patients who received an initial 7-day supply, the probability of obtaining an opioid analgesic prescription refill for nine of 10 conditions was <25%. These results suggest that a ≤7-day opioid analgesic prescription might be sufficient for most, but not all, patients seen in primary care settings with acute pain who appear to need opioid analgesics. However, treatment strategies should account for patient and condition characteristics, which might alternatively reduce or extend the anticipated duration of benefit from opioid analgesic therapy.

This analysis was based on a previously defined cohort used to characterize national patterns of prescribing for acute pain in primary care settings; details of cohort selection are described elsewhere (*4*). Briefly, adults who filled an opioid analgesic prescription within 7 days of an initial visit for any of 10 common acute pain conditions (back pain with radiculopathy, back pain without radiculopathy, neck pain, joint pain, tendon/bursal pain, muscle strains/sprains, musculoskeletal injury [e.g., ligamentous tears], urinary calculus, headache, and dental pain) evaluated in a primary care setting were identified using 2014 data from a large U.S. nationwide commercial insurer. The cohort excluded patients with history of previous opioid use, substance abuse, cancer, admission to hospice/hospital, or surgery during a baseline claims history of 6 months. Patients filling prescriptions for ≥30 days or for patch formulations also were excluded from this analysis on the assumption that, for these patients, clinicians intended to initiate long-term therapy. Patients who had <30 days of follow-up or who had multiple pain conditions or multiple opioid analgesic prescriptions associated with the index primary care visit also were excluded. Patients included in the analysis could not have more than one of the 10 acute pain conditions (i.e., only a patient’s first visit to a primary care provider that met inclusion criteria for the study was included in the analysis). Two statistical software packages were used to conduct the analyses: SAS (version 9.4; SAS Institute) and R (version 3.5.0; R Foundation for Statistical Computing).

The primary outcome of interest was opioid analgesic refills; any additional fill for oral opioid analgesics during the 30 days after the index fill was considered a refill. Refills were presumed to be an indication that the initial amount of medication prescribed was perceived as insufficient for the treatment of the patient’s pain (*5*). Descriptive statistics concerning the number of days’ supply, quantity dispensed, and morphine milligram equivalents of the index dispensing were calculated.

A logistic generalized additive model was fit for the probability of a refill as a smooth function of prescribed days’ supply separately for each condition (*5*,*6*). Models were first fit without adjustment for covariates, so that the number of days’ supply of the initial opioid analgesic fill was the only variable in the model; models were then fit with adjustment for age, sex, and Charlson comorbidity score (an index for estimating mortality from comorbid conditions in longitudinal studies) (*7*). Age was included in the model using a smooth term to allow for nonlinearity of the association between age and outcome. This model was used to estimate the probability of a refill associated with an initial supply of 3, 5, 7, 14, or 28 days for the average patient with each condition.

Among the 176,607 patients meeting selection criteria with a visit to a primary care setting for an episode of acute pain, a total of 13,440 (7.6%) filled an opioid analgesic prescription within 7 days of the initial visit; the percentage varied by condition, from 1,229 (3.5%) for headache to 302 (27.6%) for dental pain (Table 1). Among patients who filled a prescription for opioid analgesics, the median initial amount filled across conditions ranged from 4–7 days, 20–30 tablets or capsules, and 100–155 morphine milligram equivalents. A total of 2,392 (17.8%) patients who were dispensed an opioid analgesic (i.e., approximately 1% of the full cohort) obtained at least one refill within 30 days after their initial prescription. Higher unadjusted rates of refills occurred among men (19.3%) than among women (15.8%), as well as among patients with recent history of use of benzodiazepines (26.5%), sedative hypnotics (20.0%), or gabapentin (28.3%), relative to the overall refill rate (17.8%) (Table 1).

**TABLE 1:** Quantity of opioid analgesics filled after initial visits for acute pain in primary care settings, by patient characteristics — United States, 2014

Characteristic	No. of patients with visit for acute pain	No. of patients with opioid fill within 7 days of initial visit (%)	Index fill: no. of days’ supply dispensed,* median (IQR) (10th percentile) (90th percentile)	Index fill: no. of tablets/capsules dispensed,* median (IQR) (10th percentile) (90th percentile)	Index fill total MME dispensed,* median (IQR) (10th percentile) (90th percentile)	No. of patients with ≥1 refill (%)*
**Sex**						
Women	88,831	5,815 (6.5)	7 (4–10) (3–15)	30 (20–40) (15–60)	150 (100–225) (75–300)	918 (15.8)
Men	87,776	7,625 (8.7)	7 (4–10) (3–15)	30 (20–40) (15–60)	150 (100–225) (75–338)	1,474 (19.3)
**Baseline medication**						
Benzodiazepines	6,291	810 (12.9)	7 (5–10) (3–15)	30 (20–40) (15–60)	150 (120–250) (90–375)	215 (26.5)
Sedative hypnotics	4,325	375 (8.7)	7 (5–10) (3–15)	30 (21–40) (15–60)	150 (150–300) (100–480)	75 (20.0)
Gabapentinoids	1,515	187 (12.3)	8 (5–13) (4–15)	30 (30–60) (16–75)	200 (150–300) (90–450)	53 (28.3)
**Baseline Charlson comorbidity score^†^**						
0	152,669	11,680 (7.7)	6 (4–10) (3–15)	30 (20–40) (15–60)	150 (100–225) (75–300)	2,071 (17.7)
1	19,462	1,406 (7.2)	7 (5–10) (3–15)	30 (20–40) (15–60)	150 (120–300) (100–400)	256 (18.2)
2	3,533	279 (7.9)	8 (5–12) (3–15)	30 (30–50) (20–60)	200 (150–300) (100–450)	48 (17.2)
≥3	943	75 (8.0)	8 (5–11) (4–15)	30 (20–50) (15–60)	150 (150–300) (100–450)	17 (22.7)
**Pain conditions** ^§^						
Joint pain	56,474	2,761 (4.9)	7 (5–10) (3–15)	30 (20–40) (15–60)	150 (113–250) (80–400)	521 (18.9)
Back pain without radiculopathy	41,862	5,602 (13.4)	7 (5–10) (3–15)	30 (20–40) (15–60)	150 (100–225) (75–300)	922 (16.5)
Headache	34,718	1,229 (3.5)	6 (4–10) (3–15)	30 (20–40) (12–100)	150 (100–240) (75–600)	144 (11.7)
Neck pain	11,943	1,101 (9.2)	7 (4–10) (3–15)	30 (20–40) (15–60)	150 (100–225) (75–300)	216 (19.6)
Tendonitis/Bursitis	13,371	457 (3.4)	7 (4–10) (3–15)	30 (20–40) (15–60)	150 (100–225) (75–300)	81 (17.7)
Muscular strains/Sprains	9,034	812 (9.0)	5 (3–7) (2–10)	20 (20–30) (12–42)	120 (100–150) (75–300)	132 (16.3)
Back pain with radiculopathy	3,925	684 (17.4)	7 (5–10) (3–15)	30 (20–40) (15–60)	150 (120–225) (100–300)	203 (29.7)
Nephrolithiasis	2,980	422 (14.2)	5 (3–8) (2–10)	26.5 (20–30) (15–50)	150 (100–225) (75–300)	81 (19.2)
Musculoskeletal injury	1,205	70 (5.8)	7 (4–10) (3–15)	30 (20–40) (15–60)	155 (125–225) (95–425)	21 (30.0)
Dental pain	1,095	302 (27.6)	4 (3–6) (2–10)	20 (15–30) (12–30)	100 (75–150) (60–225)	71 (23.5)

**Abbreviations:** IQR = interquartile range; MME = morphine milligram equivalents.

* Among patients with at least one fill for opioids for an episode of acute pain.

**^†^** An index for estimating mortality from comorbid conditions in longitudinal studies. https://www.ncbi.nlm.nih.gov/pubmed/3558716.

^§^ Additional detail regarding *International Classification of Diseases, Ninth Revision* codes used to define conditions is available at https://www.ncbi.nlm.nih.gov/pubmed/28971545.

The adjusted probability of a refill appeared to decrease with increasing initial prescription duration for some conditions (e.g., back pain with radiculopathy, nephrolithiasis, or dental pain), whereas for other conditions, the adjusted probability of a refill remained relatively constant regardless of the amount initially prescribed (e.g., joint pain or nonradicular back pain) (Table 2). For an initial prescription of 7 days, the adjusted probability of refill ranged from 0.11 (95% confidence interval = 0.09–0.14) for headache to 0.41 (0.19–0.68) for musculoskeletal injury (Table 2).

**TABLE 2:** Crude and adjusted* probabilities of refill by number of days initially supplied and acute pain condition

Condition	No. of days initially supplied (95% CI)				
	3	5	7	14	28
**Joint pain**					
Crude	0.20 (0.18–0.23)	0.20 (0.18–0.22)	0.20 (0.18–0.22)	0.18 (0.16– 0.21)	0.12 (0.05–0.24)
Adjusted	0.20 (0.17–0.22)	0.20 (0.18–0.22)	0.20 (0.18–0.22)	0.18 (0.16–0.22)	0.12 (0.05–0.25)
**Back pain without radiculopathy**					
Crude	0.17 (0.16–0.19)	0.17 (0.16–0.18)	0.16 (0.15–0.17)	0.15 (0.13–0.17)	0.12 (0.09–0.17)
Adjusted	0.16 (0.14–0.18)	0.16 (0.14–0.17)	0.15 (0.14–0.17)	0.14 (0.12–0.16)	0.11 (0.08–0.16)
**Headache**					
Crude	0.14 (0.11–0.17)	0.13 (0.11–0.15)	0.12 (0.097–0.14)	0.10 (0.07–0.15)	0.12 (0.03–0.38)
Adjusted	0.13 (0.10–0.16)	0.12 (0.093–0.14)	0.11 (0.086–0.14)	0.09 (0.07–0.14)	0.10 (0.02–0.31)
**Neck pain**					
Crude	0.22 (0.18–0.25)	0.21 (0.18–0.24)	0.20 (0.18–0.22)	0.17 (0.13–0.22)	0.13 (0.06–0.26)
Adjusted	0.25 (0.20–0.30)	0.23 (0.19–0.28)	0.22 (0.19–0.26)	0.19 (0.14–0.25)	0.13 (0.06–0.26)
**Tendonitis/Bursitis**					
Crude	0.17 (0.12–0.22)	0.17 (0.13–0.21)	0.17 (0.14–0.21)	0.18 (0.12–0.26)	0.21 (0.07–0.48)
Adjusted	0.17 (0.12–0.23)	0.17 (0.13–0.22)	0.18 (0.14–0.22)	0.19 (0.12–0.27)	0.21 (0.07–0.49)
**Muscular strains/Sprains**					
Crude	0.17 (0.13–0.20)	0.16 (0.13–0.19)	0.15 (0.12–0.18)	0.13 (0.07–0.21)	0.09 (0.02–0.31)
Adjusted	0.16 (0.13–0.20)	0.16 (0.13–0.19)	0.15 (0.12–0.18)	0.13 (0.07–0.22)	0.09 (0.02–0.32)
**Back pain with radiculopathy**					
Crude	0.31 (0.24–0.39)	0.33 (0.27–0.40)	0.28 (0.21–0.35)	0.21 (0.12–0.34)	—^†^
Adjusted	0.24 (0.16–0.35)	0.27 (0.20–0.36)	0.21 (0.14–0.30)	0.15 (0.07–0.29)	—^†^
**Nephrolithiasis**					
Crude	0.21 (0.16–0.26)	0.20 (0.16–0.25)	0.20 (0.15–0.25)	0.18 (0.08–0.35)	0.15 (0.02–0.61)
Adjusted	0.22 (0.15–0.31)	0.22 (0.15–0.29)	0.21 (0.14–0.30)	0.19 (0.08–0.39)	0.16 (0.02–0.65)
**Musculoskeletal injury**					
Crude	0.26 (0.12–0.47)	0.49 (0.27–0.72)	0.37 (0.18–0.62)	0.41 (0.13–0.77)	—^†^
Adjusted	0.21 (0.086–0.44)	0.55 (0.30–0.79)	0.41 (0.19–0.68)	0.48 (0.15–0.84)	—^†^
**Dental pain**					
Crude	0.27 (0.21–0.33)	0.23 (0.19–0.29)	0.20 (0.14–0.28)	0.12 (0.04–0.31)	0.04 (0.003–0.39)
Adjusted	0.27 (0.21–0.33)	0.23 (0.18–0.29)	0.20 (0.14–0.28)	0.12 (0.04–0.31)	0.04 (0.003–0.39)

**Abbreviation:** CI = confidence interval.

* Adjusted for age, sex, and Charlson comorbidity score. (https://www.ncbi.nlm.nih.gov/pubmed/3558716). Probabilities calculated for the “average patient.”

^†^ Estimate is not informative because of sparse data. Twenty-eight days’ supply estimates are outside the range of data for most conditions.

## Discussion

These findings, drawn from the claims of nationwide commercial insurance beneficiaries, indicate that in 2014 the median duration of initial opioid analgesic prescriptions for acute pain indications in a primary care setting was 4–7 days. Fewer than one in five patients who filled an opioid analgesic prescription received a refill, suggesting that in most cases an initial prescription of this duration was considered sufficient (and possibly even more than necessary) for patients seen in primary care settings with acute pain, consistent with recommendations in the 2016 CDC Guideline for Prescribing Opioids for Chronic Pain (*8*). These results also suggest that providing a 7-day supply might risk overtreatment for some of these conditions. However, depending upon the specific condition, the probability of receiving a refill after an initial 7-day supply ranged from 0.11–0.41, underscoring the potential variation among patients in time to recovery and variation in clinician practice, as well as possible variation in availability of nonopioid treatment methods. Because legal limits on prescribing are imposed despite such variation, these results suggest that health systems will need to be equipped to provide efficient mechanisms for opioid analgesic refills when they are clinically appropriate (*9*,*10*).

The findings in this report are subject to at least four limitations. First, this analysis preceded the implementation of many prescribing limits on opioids (*2*,*3*). Accordingly, compared with filling behaviors observed during the period assessed by this study (i.e., 2014), observed filling behaviors in more recent years might be distinct, potentially influenced by factors external to the patient-physician interaction, including policies enforced by states, health systems, or private stakeholders. Second, although absence of a refill was used as a surrogate for adequacy of the initially dispensed supply for controlling pain, in addition to a need for additional opioid therapy, opioid refills might reflect physical dependence, withdrawal, or the need for additional pain control that could possibly be managed by nonopioid alternatives. Third, refills within 30 days of an initial opioid fill were presumed to be for treatment of the same pain condition as the initial fill, although given the lack of direct linkage between the diagnostic and prescription claims, this assumption could not be verified. Finally, certain patient characteristics, such as male sex and recent use of benzodiazapenes, were associated with higher refill rates; however, these associations should be interpreted with caution because these factors might be associated with conditions requiring longer duration of treatment rather than being independent risk factors for additional fills. Although efforts were made to stratify analyses on distinct pain etiologies targeted by opioid analgesic prescribing in the primary care setting, some heterogeneity with respect to etiology, duration, and severity of pain within these categories is likely.

Future research could aim to further clarify the natural history of acute pain across a range of settings and conditions and to identify the risks and benefits of opioid analgesic use for acute pain through in-depth prospective interviews with patients. Simultaneously, research to evaluate the impact of recent opioid analgesic prescribing guidelines upon patient-centered outcomes including, but not limited to, adequacy of pain control, misuse of opioids, or development of opioid use disorder, is needed. Such measures might help determine whether existing strategies to regulate opioid analgesic prescribing result in an acceptable benefit-to-harms ratio. The findings in this report suggest that for several acute pain conditions evaluated in primary care settings, opioid analgesics, when provided to treat pain, can generally be prescribed for durations of ≤7 days. However, health systems must anticipate variation in patient, condition, and other contextual characteristics that will influence the duration and intensity of pain and adopt mechanisms to ensure that additional access to both pharmacologic and nonpharmacologic therapy is available when required.

**Summary**

**What is already known about this topic?**

The prescribed duration of opioid analgesics for acute pain in the primary care setting varies by patient and condition.

**What is added by this report?**

For 10 acute pain conditions commonly managed in primary care settings, the probability of obtaining a refill after an initial 7-day opioid analgesic prescription ranged from 11% (headache) to 41% (musculoskeletal injury), with refill probability <25% for most conditions.

**What are the implications for public health practice?**

Initial opioid analgesic prescriptions of ≤7 days’ duration appear sufficient for many patients seen in primary care settings with acute pain. Treatment strategies should account for patient- and condition-specific characteristics, which might reduce or extend duration of benefit from opioid analgesic therapy.
